# A shift in women’s health? Older workers’ self-reported health and employment settings during the COVID-19 pandemic

**DOI:** 10.1093/eurpub/ckab204

**Published:** 2021-11-27

**Authors:** Jacques Wels, Natasia Hamarat

**Affiliations:** 1 MRC Unit for Lifelong Health and Ageing, Faculty of Population Health Sciences, University College London, London, UK; 2 METICES Centre & Centre de Droit Public, Université libre de Bruxelles, Brussels, Belgium

## Abstract

**Background:**

The first wave of COVID-19 has had a massive impact on work arrangements settings in many European countries with potential effects on health that are likely to vary across gender.

**Methods:**

Focusing on the workforce aged 50 and over in 27 European countries using data from SHARE wave 8 (N = 11,221), the study applies a generalized logit mixed-effects model to assess the relationship between negative and positive change in self-reported health since the start of the pandemic and change in employment settings using an interaction effect between gender and employment arrangements to distinguish their specific association by gender after controlling for socio-economic covariates and multicollinearity.

**Results:**

Female respondents have higher probabilities to declare a positive health when working fully or partially from home or when temporarily and permanently unemployed. However, introducing the main effect of gender exacerbates discrepancies and such benefits fade away. Differences across countries do not significantly change the estimates.

**Conclusion:**

The benefits of work arrangements to improve women’s health during the first wave of COVID-19 have not compensated the negative effect of gender discrepancies exacerbated by the pandemic to the extent that employment arrangements have no role, or just a negative impact, in modulating them.

## Introduction

The COVID-19 pandemic and the measures that have been taken to control its spread in Europe have had a massive impact on the economy and the labour market. On the one hand, the pandemic has caused job losses with particular negative effects on women’s employment participation^1^ that have varied based on the type family compositions and living conditions.^2^ On the other hand, the pandemic has affected the way employment is organized, with an increasing use of flexible forms of employment including part-time work^3^ and working from home^4^ and the implementation of temporary employment schemes commonly called ‘furlough’.^5^ In this context and given the specific nature of the crisis,^6^ the role played by employment arrangements in explaining older workers’ health is still to be investigated.

It is well known that the employment status—and, more precisely, transitions over different employment statuses—is associated with health outcomes. A large corpus of studies has underlined the negative impact of unemployment on physical and mental health,^7–9^ wellbeing^10^ and risk of death,^11^ and employment is usually perceived as positive when looking across health discrepancies across the population. But such an impact varies across gender—women’s self-reported and mental health is more negatively affected by unemployment than men.^12^

However, the relationship between employment and health has to be balanced as job insecurity also has detrimental effects^13^ and health is also seen as a predictor of unemployment (selection hypothesis) and not only as an outcome (causation hypothesis).^14^ Uncertainty being a key factor in understanding the effect of unemployment on health,^13^ the impact of furlough is not well known. Some studies have demonstrated, in some very particular contexts, the positive role played by temporary unemployment in comparison with permanent unemployment^15^ but neither the impact of temporary unemployment on older workers’ health nor the interaction between gender and furloughing have been explored so far.

Aside from the distinction between employment and unemployment, the current pandemic is also characterized by the development of less-standard forms of employment among which working time reduction and working from home have played a preponderant role. Working time is known as being a key factor in explaining health variations among the workforce^16^ but the voluntariness or involuntariness of change in working time^17^ such as the policies that allow for working time regulations and the arrangements that compensate the income loss after reducing working time^18^ play a role. This also applies to the ageing workforce with particular positive health effects on low-income workers.^19^ The relationship between working from home and health is more ambiguous. In the current context, teleworking has not been anticipated by workers or employers with potential effects on occupational health.^20^ Additionally, remote work is sometimes associated with overlapping responsibilities such as domestic labour and childcare.^21^ Some recent contributions argue that the lockdown(s) might have represented an opportunity to reduce the gender gap, especially because companies must adopt flexible working arrangements^22^ and men have increased the amount of time devoted to housework.^23^ But other studies demonstrate that gender discrepancies have been exacerbated by the pandemic.^24^ Little attention has been paid so far to the effects on health, especially regarding the specific case of the older workforce.^25^

Several research perspectives that have to be addressed flow from this background. First, the relationship between employment status and health has to account for a gradient of statuses and transitions. Opposing employment and unemployment would oversimplify the set of individual situations that can be observed since the start of the pandemic. The COVID-19 crisis has caused permanent job loss, but temporary unemployment has been common across Europe. Similarly, continuous employment has changed in nature as many workers have moved from a traditional workplace setting to a more complex work organization including changes in working time and home working. Second, women have been more affected by certain types of statuses (e.g. unemployment) than men, which might introduce a selection bias when looking at the association between employment status and health. Similarly, changes in employment statuses may have affected women’s health in different ways. Third, the impact of the pandemic goes beyond employment statuses, and one has to account for the gender differences in health that are not explained by labour market settings.

The purpose of this study is to offer empirical evidence about these perspectives. Using data from the recently released wave eight of the Survey of Health, Ageing and Retirement (SHARE), the study investigates the relationship between employment arrangements and self-reported change in health following the first wave of COVID-19 in Europe of workers aged 50 and over, distinguishing the effects of these arrangements on self-reported health, on the one hand, and how these effects vary across gender, on the other hand. In other words, the study questions the respective role of employment arrangements and gender and how their interaction results in different self-reported health outcomes.

## Methods

### SHARE wave 8

The study uses micro-data from SHARE,^26^^,^^27^ wave 8. Data collection for wave 8 was planned to start in late 2019 but the spread of COVID-19 in February 2020 has changed the original plan.^28^ Instead, it was decided to carry one with follow-up phone interviews with a questionnaire specifically dedicated to the pandemic situation that includes questions about different aspects including health, safety and work and employment conditions. The current dataset is an early beta release containing data collected via computer-assisted telephone interviews between June and August 2020. The sample design strategy has previously been described by Scherpenzeel et al.^28^ This study uses data for all the 27 countries included in the survey. The original sample size was 70 275 and was reduced to 11 221 after removing respondents who were not employed prior the start of the pandemic. All respondents included in the sample had, at least, participated in one of the previous waves.

The selected sample includes respondents aged 50 and over who declared being employed or self-employed prior the start of the pandemic, independently from their post-pandemic status and for whom retrospective data about employment trajectories, education level and number of children were available either in wave 3 or 7 with a number of missing values of 1622. Countries are not represented in the same way in the dataset, ranging from 12.2% of the sample in Estonia to 1.5% in Spain in the final sample after selection.

### Self-reported change in health

Wave 8 contains two main information about self-perceived health (SPH). Respondents were asked what was their SPH prior the start of the pandemic (five modalities, from excellent to poor) and how their SPH had changed since the outbreak of COVID-19 (in three modalities, i.e. worse, better or same). The study looks up at whether SPH has worsened since the start of the pandemic distinguishing, on a binary basis, those who reported a worsened health from those who reported the same or a better SPH (reference category). The model accounts for pre-pandemic SPH as a control variable.

SPH-types variables are largely discussed in the literature on, at least, two aspects. First, the variable requires an in-depth understanding of its distribution features because, as calculated on a Likert scale, it could take the form of a Poisson distribution.^29^ As the variable contains only three modalities, the choice was made to use it as a binary variable by distinguishing those who experienced a negative change in SPH from those who did not. Second, the association between SPH and other health indicators such as the reliability of SPH when working with panel data has been discussed. On the one hand, an important corpus of studies has demonstrated that SPH could be a predictor of mortality that is independent of objective heath statuses.^30^ But, on the other hand, the reliability of the self-assessed health status can also be questioned, particularly in the context of repeated measurements^31^ as the change in response over time largely depends on the socio-economic group and age but could also be affected by cross-national differences when using comparative data.^32^

### Change in employment status

The model pays particular attention to gender and employment transitions. In this study, gender is coded on a binary basis using ‘male’ as the reference category. Employment transitions are more complex because they are based on a classification using the type of employment status, the type of employment setting and potential changes in working time. The employment status distinguishes those who fully lost their job (unemployment) from those who lost their job temporarily (partial unemployment). They both account for 18% of the sample (table 1). However, no information was collected on whether partial unemployment was furloughed or on whether respondents transitioned to another job. For those who worked during the data collection period (between June and August 2020), data were collected about change in working time (higher, lower or same) and employment settings (workplace, home working or both). Nine categories were created using these two dimensions.

**Table 1:** Employment arrangements by gender, descriptive statistics

	Dataset				Matched dataset			
	Male	Female	Percentage of female	Total percentage	Male	Female	Percentage of female	Total percentage
Unemployment	475	702	0.60	0.10	152	187	0.55	0.08
Partial unemployment	418	504	0.55	0.08	150	121	0.45	0.07
Partial home working/higher	90	128	0.59	0.02	35	29	0.45	0.02
Partial home working/lower	126	150	0.54	0.02	38	54	0.59	0.02
Partial home working/same	737	915	0.55	0.15	230	233	0.50	0.11
Home working/higher	89	227	0.72	0.03	25	65	0.72	0.02
Home working/lower	138	206	0.60	0.03	38	54	0.59	0.02
Home working/same	387	567	0.59	0.09	119	142	0.54	0.06
Workplace/higher	194	271	0.58	0.04	80	70	0.47	0.04
Workplace/lower	315	353	0.53	0.06	114	103	0.47	0.05
Workplace/same	2.092	2.034	0.49	0.37	1006	1047	0.51	0.50
Other	50	53	0.51	0.01	20	11	0.35	0.01
Total	5.111	6,110		1.00	2,007	2,116		1.00
Mean			0.57				0.52	

Notes: SHARE wave 8, authors’ calculation.

### Covariates

The model includes several covariates. A quadratic function of age; pre-pandemic SPH; the number of children at 50 distinguishing no child (reference category), one child, two children and three or more children (no children being the reference category); self-reported net household income prior the pandemic; and the ratio (in percentage) between self-reported net household income prior and after the pandemic. The model also controls for the direct impact of COVID-19. SHARE contains two information about this: whether respondents were tested positive and whether they reported COVID-19-related symptoms, independently of whether they were contaminated or tested. As the study looks at SPH and as asymptomatic cases are frequent, one variable is included that distinguishes those who reported COVID-19 symptoms from those who did not. The model also controls for employment trajectories prior the pandemic. Sequence analysis was performed using four possible statuses along the career: unemployment, retirement, education, full-time work and part-time work.^33^ By doing so, employment trajectories are distinguished depending on whether they were characterized by a stable or changing working time. The distance between the sequence clusters was calculated using optimal matching methods^34^ with 9520 sequences containing 3963 distinct sequences. Four clusters flew from the sequence analysis: early education exit with full-time career (cluster 1); late education exit with full-time career (cluster 2); part-time career (cluster 3); multiple employment transitions (cluster 4).

### Models

The model used in this study is a generalized logit mixed-effects model for binary outcomes that is a multilevel modelling allowing random intercept and slopes.^19^ The model is replicated twice.

Model 1 sets up a random intercept based on country-units and a random slope for employment arrangements. The random slope for a categorical independent variable is the random difference at the intercept and allows fixed-effects employment arrangements to vary by country. The formula for model 1 is:

$${\left(\mathrm{model}\;1\right)\;Y}_{ij}={\;\beta }_{0}+{\;(\beta }_{1}+\cup_{1is})X_{1ij}+{\;\beta }_{c}C_{ij}+\;\cup_{0j}+\varepsilon_{0ij}\;\;\;\;\;\;={\;\beta }_{0}+{\;\beta }_{1}X_{1ij}+{\;\beta }_{c}C_{ij}+\;\cup_{0j}+\cup_{1j\;}{X_{1ij}+\varepsilon }_{0ij}$$

where a random slope ‘U_is_’ is introduced to allow differences in slope across countries for the variable of interest ‘X’ (Types of employment arrangements).

Model 2 replicates model 1 on a matched dataset. As the models assess the relationship between a set of independent variables including gender, work and employment arrangements and income and the change in health since the outbreak of COVID-19, collinearity between the independent variables is a possibility. Put in another way, gender, incomes and health prior the pandemic could partially explain employment transitions following the virus outbreak. To control for this, a matched dataset was created using propensity score matching methods.^35^ The matching was calculated using a nearest neighbour matching selection^36^ based on the propensities of moving to non-standard forms of employment versus working within the workplace and keeping the same working time. The set of independent variables was composed of gender, age, SPH prior the pandemic, number of children, type of employment trajectory and household net income prior the pandemic. Both models include normalized population weights.

Models 3 and 4 replicate model 1 and 2 using inverse probability of attrition weights calculated as the inverse probability of being included in wave 8 whilst being in one or several previous waves. The inverse probability was calculated using age, gender, pre-pandemic and current SPH, employment status and whether respondents had COVID-19-related symptoms as the explanatory variables.

Finally, to test for the relationship between employment status and gender, results from models 1–4 were replicated including an interaction effect. As the model is in logit, predicted probabilities were calculated. Two types of probabilities were calculated. P_1_ looks up at the difference in probability (to declare a worse SPH)—i.e. the *antilogits—*between men and women taking into account the interaction effect of work and employment arrangements by gender but excluding the main effect of gender. P_2_ replicates the differences in probabilities but includes the gender main effect. By doing so, the model distinguishes the specific impact of employment arrangements by gender excluding the impact of gender that is not related to employment arrangements (P_1_) and the impact of employment arrangements combined with the impact of gender independently from employment settings.

## Results

### Descriptive statistics

Table 1 exhibits descriptive statistics for work and employment arrangements by gender, the percentage of female within each employment transition and the total percentage of transitions among the workforce in the original and the matched datasets. What can be observed is that those working within the workplace and keeping the same working time, respectively, account for 37 and 51% of the sample of the original and matched datasets. The second type of arrangements is partial home working (i.e. the combination of workplace and home working) with no change in working time (same) as they account for 15% and 11% of the sample again equally distributed across gender. Discrepancies occur when looking at unemployment and home working with higher working time.

### Propensity scores

**Figure 1:**
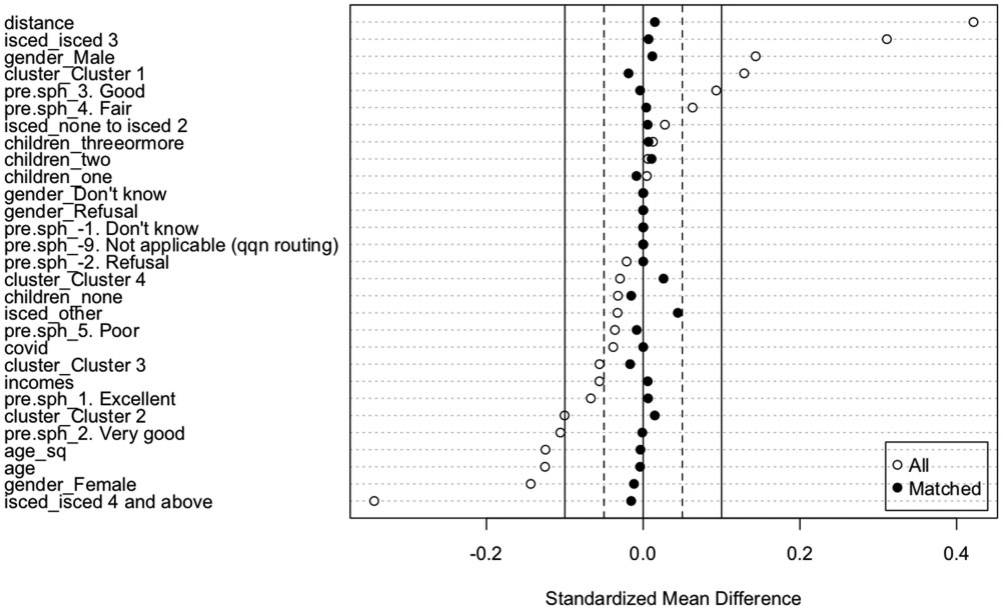
Propensity scores

Figure 1 exhibits the propensity scores as the standardized mean differences in the full and the matched datasets. These are the propensities of being employed and work within the workplace with no change in working time. What figure 1 shows is that male workers, those who have a stable full-time career (cluster 1) and those with intermediary levels of education (ISCED 3) have had higher propensities of keeping working within the workplace with no change in working time. By contrast, changes in employment status were more associated with being a female, being highly educated respondents (ISCED 4 and above) and having had a part-time career.

### Main effect

Table 2 shows the results, in odds ratios, of models 1–4. Four main observations pop out regarding the variables of interest. First, partial home working is associated with higher odds of declaring a worsening health when associated with a change in working time (either higher or lower) is observed. Odds are significant at 99% in models 1–3 and non-significant in model 4. Conversely, partial home working and similar working time seems that have detrimental effects (only significant in model 3). Second, odds below 1 are also observed for home working. These are significant in most models when working time is the same or has increased, no statistically significant result is observed for those who reduced working time. Third, results indicate a potential negative relationship between SPH and unemployment and a potential positive relationship between SPH and partial unemployment, but estimates are not significant. Finally, the impact of gender is consistent across models: being a female multiplies the odds of declaring a worsening SPH by two.

**Table 2:** Generalized logit mixed-effects model

		Model 1		Model 2		Model 3		Model 4	
		OR	95% CI	OR	95% CI	OR	95% CI	OR	95% CI
	**Variables of interest**								
	Unemployment	1.58	[0.69–3.65]	1.27	[0.60–2.73]	1.56	[0.66–3.66]	1.80^.^	[0.94–3.46]
	Partial unemployment	0.96	[0.34–2.71]	0.89	[0.31–2.54]	0.98	[0.35–2.76]	0.41	[0.13–1.34]
	Part home working/higher	0.06^***^	[0.02–0.21]	0.03^***^	[0.01–0.13]	0.06^***^	[0.02–0.21]	0.36	[0.13–1.02]
	Part home working/lower	0.00^***^	[0.00–0.04]	0.00^***^	[0.00–0.04]	0.00^***^	[0.00–0.04]	0.00	[0.00–Inf]
	Part home working/same	1.25	[0.65–2.42]	1.14	[0.61–2.16]	1.31^***^	[0.67–2.58]	1.02	[0.36–2.89]
	Home working/higher	0.28^***^	[0.09–0.89]	0.17^*^	[0.04–0.68]	0.30^**^	[0.10–0.93]	0.30	[0.07–1.29]
	Home working/lower	0.57	[0.15–2.11]	0.48	[0.16–1.49]	0.57	[0.16–2.04]	0.14	[0.06–1.57]
	Home/same	0.40^**^	[0.19–0.85]	0.31^**^	[0.14–0.69]	0.41^***^	[0.19–0.86]	0.24^**^	[0.07–0.82]
	Workplace/lower	0.26^*^	[0.09–0.80]	0.25^*^	[0.09–0.73]	0.44^**^	[0.20–0.95]	0.04	[0.01–0.24]
	Workplace/higher	0.42^**^	[0.20–0.91]	0.29^*^	[0.11–0.77]	0.26^*^	[0.08–0.82]	0.02^**^	[0.00–0.16]
	Other	0.00^***^	[0.00–0.06]	0.00^***^	[0.00–0.06]	0.00	[0.00–0.09]	0.00^***^	[0.00–0.02]
	Gender: female	2.04^***^	[1.82–2.29]	2.01^***^	[1.79–2.25]	2.05^***^	[1.82–2.29]	2.72^***^	[1.36–3.12]
	**Covariates**								
(1)	SPH prior: excellent	0.60^***^	[0.49–0.73]	0.58^***^	[0.48–0.71]	0.58^***^	[0.48–0.71]	0.83^*^	[0.67–1.04]
	SPH prior: very good	0.52^***^	[0.45–0.60]	0.52^***^	[0.45–0.60]	0.51^***^	[0.44–0.58]	0.44^***^	[0.36–0.53]
	SPH prior: fair	2.05^***^	[1.79–2.35]	2.23^***^	[1.95–2.55]	2.06^***^	[1.80–2.37]	2.26^**^	[1.93–2.64]
	SPH prior: poor	1.94^***^	[1.47–2.56]	2.00^***^	[1.51–2.64]	1.97^***^	[1.49–2.61]	1.48^**^	[1.03–2.13]
(2)	Covid symptoms	7.61^***^	[6.53–8.87]	6.81^***^	[5.81–7.98]	7.83^***^	[6.70–9.14]	8.70^***^	[7.21–10.49]
(3)	Age	1.43^***^	[1.21–1.70]	1.39^***^	[1.17–1.65]	1.42^***^	[1.19–1.69]	1.38^***^	[1.13–1.67]
	Age square	1.00^***^	[1.00–1.00]	1.00^***^	[1.00–1.00]	1.00^***^	[1.00–1.00]	1.00^***^	[1.00–1.00]
(4)	1 Child	0.58^***^	[0.48–0.69]	0.60^***^	[0.50–0.71]	0.58^***^	[0.48–0.70]	0.58^**^	[0.46–0.72]
	2 Children	0.86^**^	[0.75–1.00]	0.90	[0.78–1.05]	0.87	[0.75–1.02]	0.81^***^	[0.68–0.98]
	3 Children or more	1.23^**^	[1.05–1.44]	1.18^*^	[1.01–1.39]	1.25^***^	[1.07–1.46]	1.41^***^	[1.16–1.71]
(5)	None to ISCED 2	0.97	[0.83–1.14]	0.91	[0.77–1.07]	0.99	[0.85–0.16]	1.34^**^	[1.11–1.60]
	ISCED 4 and above	1.14^***^	[1.02–1.29]	1.59^***^	[1.32–1.91]	1.15^***^	[1.02–1.29]	1.40^***^	[1.21–1.61]
(6)	Cluster 2	1.08	[0.94–1.23]	1.03	[0.90–1.17]	1.06^.^	[0.93–1.21]	1.21^**^	[1.03–1.42]
	Cluster 3	1.39^***^	[1.17–1.63]	1.38^***^	[1.17–1.63]	1.39^***^	[1.17–1.64]	1.29^***^	[1.29–1.59]
	Cluster 4	0.67^***^	[0.56–0.81]	0.52^***^	[0.43–0.64]	0.67^***^	[0.56–0.81]	0.48^***^	[0.38–0.60]
(7)	Net incomes prior	1.06^*^	[0.97–1.16]	1.07	[0.97–1.17]	1.06	[0.97–1.17]	1.08	[0.97–1.20]
	Ratio prior/post	0.76^***^	[0.67–0.85]	0.69^***^	[0.60–0.80]	0.77^***^	[0.69–0.87]	0.70^***^	[0.61–0.81]

Source: SHARE waves 7 and 8, authors’ calculation. Note: Significance levels as follows: * p < 0.10, ** p < 0.05, *** p < 0.01. Those who kept working the same working time and kept working within the usual workplace are selected as the reference category. The reference for gender is ‘male’, (1) pre-pandemic self-perceived health (SPH) (retrospective). The reference is ‘good’; (2) respondents who declared having COVID-19 symptoms (independently of whether they were tested or not). The reference category is ‘no symptoms’; (3) quadratic function of age; (4) number of children at 50—the reference is ‘no children’; (5) level of education based on the ISCED (International Standard Classification of Education) nomenclature—the reference is ‘ISCED 3’; (6) clusters flowing from the Sequence Analysis, ‘cluster 1’ is the reference category; (7) declared total household income after tax and social contributions prior the start of the pandemic and declared change in income as a ratio between prior and post-pandemic household net income.

The set of covariates also is of interest. Pre-pandemic SPH is associated with self-reported change in SPH with significantly higher odds of declaring a negative change for those having a pre-pandemic fair or poor SPH. The level of education shows a different pattern than what could have been expected as higher levels have higher odds to declare a negative change in SPH, but this might be due to the fact that the model controls for employment pathways. Interestingly, part-time employment histories (cluster 3) increase the odds of declaring a negative change in SPH whilst interrupted employment histories (cluster 4) reduce the odds compared with stable employment pathways (cluster 1, reference category). Not surprisingly, change in incomes (i.e. the ratio between pre- and post-pandemic incomes) is significantly associated with self-reported change in health.

### Interaction effect

**Figure 2:**
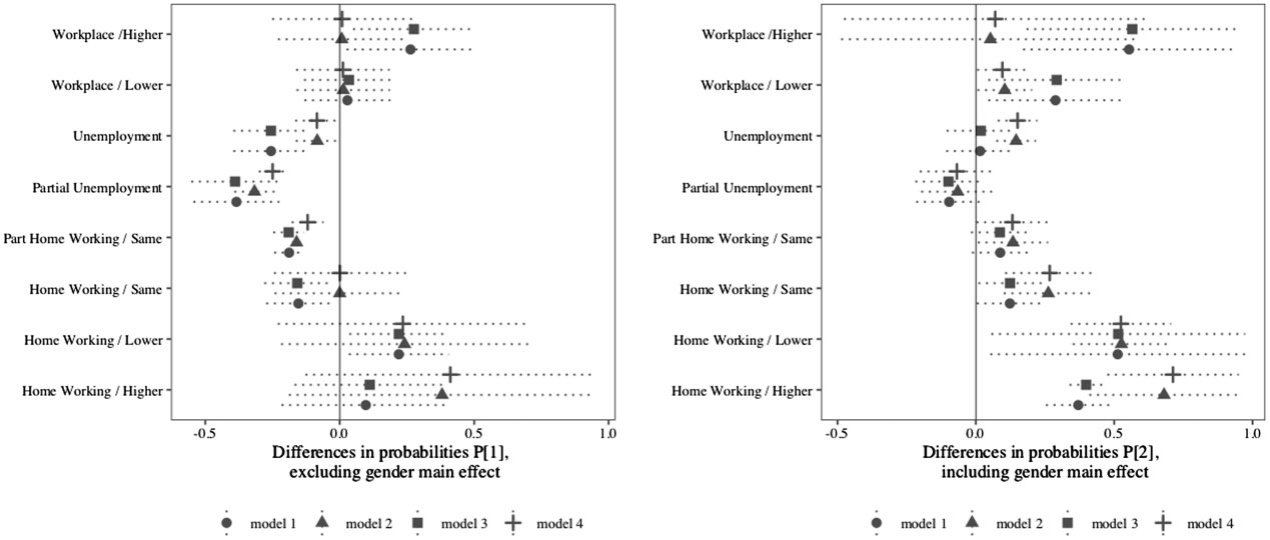
Differences in probabilities

Therefore, the question is to know how employment arrangements and gender interact. Figure 2 answers this question by calculating the predicted probabilities P_1_ (excluding gender main effect) and P_2_ (including gender main effect). The differences in probabilities excluding the gender main effect (P_1_) show that unemployment, partial unemployment, partial home working with same working time and home working with the same working time (only when using the matched dataset) are positively associated with women’s self-perceived change in health compared with men. By contrast, increasing working time with no change in employment setting and reducing working time whilst working from home have had detrimental effects (only models 2 and 4 are significant).

However, one must assume that work is not the only explanation in understanding heath discrepancies across gender. That is the reason why the right-hand side of figure 2 includes the main effect of being a female (i.e. what being a female adds up to the probabilities of declaring a negative change in SPH independently from the type of work and employment setting).

Estimates clearly indicates that the main effect of gender cancels out the positive associations that are observed for employment settings such as home working and partial home working but also the positive associations that were observed for unemployment and partial unemployment. In other words, unemployment is positively associated with women’s health (women had lower probabilities to declare a negative change in SPH) but including the main effect reduces these probabilities, which means that, even though women have beneficiated from unemployment in a way, other factors have neutralized such a positive relationship.

### Limitations

The study contains several limitations that will be partially addressed when further waves are released. First, the study does not use proper longitudinal data as it is based on the use of retrospective data both about health prior and after the pandemic outbreak. Second, the survey does not directly distinguish partial from permanent unemployment. Even though the way questions were asked allows to distinguish those who were unemployed since the outbreak of the virus to the interview time from those who had work activities, there is a lack of information about the type of employment scheme that was used. Similarly, no question was asked about potential retirement plans whilst the current situation could contribute to pushing older workers to retire earlier than expected.^37^ Third, the country response to COVID-19 such as the percentage of infection was diversified across Europe. One faces different epidemiologic settings with different types of work and employment arrangements that cross-national comparison, based on a limited amount of information (at this stage), cannot control. Fourth, the dataset does not contain clear information about the nature of the work that is actually done, nor does it include information about sectors of activity. Fourth, no question was asked about care activities, particularly for parents, children and grandchildren whist grandparenthood and care for a relative have detrimental effects on health, particularly for women.^38^ Fifth, various financial supports were implemented in Europe that are not included in this study as the detail about the nature of these arrangements is not available in SHARE. Finally, changes in employment settings might be associated with changes in social contacts that the study was not able to assess.

## Discussion

These results question health discrepancies in Europe during the COVID-19 crisis. Self-reported health is usually not associated with major differences across gender.^39^ Going back to some descriptive statistics illustrates the current situation: self-reported health prior the pandemic was reported equally across gender, with 47% and 27% of workers, respectively, reporting a very good or fair health in both the female and the male samples with a difference of <1% when looking at poor or excellent health. What the study shows is that women have had higher odds of reporting a negative change in SPH since the start of the pandemic, independently from any kind of COVID-19-related symptoms.

Employment arrangements implemented during the first wave of COVID-19 in Europe partially explain gender discrepancies when looking at older workers’ change in self-reported health. However, they do not, as such, explain these differences and other gender-related aspects are associated with a negative change in SPH. This suggests that the increasing use of less-standard forms of employment including, particularly, home working would not lead to a more gender-equal society as expected by some authors.^22^^,^^40^

Three major results flow from the study that have policy implications.

First, the study shows that unemployment and temporary unemployment are positively associated with women’s self-reported health. Second, (partial) home working also has a positive impact on self-reported change in health. What flows from the study is that both changes in employment settings and temporary or permanent unemployment are preferable to keeping working within the usual workplace. However, a large part of the ageing workforce has not had access to these schemes, and the question is therefore to know how one could reduce the adverse health effects of continuous employment settings. Thirdly, even though women’s health has beneficiated from home working, partial home working and unemployment, other gender-related factors have cancelled out these beneficial effects. A major policy issue is therefore to focus on the out-of-employment factors that have played a negative role in explaining women’s self-reported health—including social contacts and social isolation, access to health care services, housing quality or social benefits and incomes, to name just a few—and that could have negatively interacted with the employment policies that were implemented during the crisis.

## Funding

The authors received no specific funding for this work.

*Conflicts of interest*: None declared.

Key points

- 50+ female workers have reported higher odds of being affected by a negative change in self-reported health since the start of the COVID-19 compared with men.
- Home working and working time reduction are associated with positive change in women’s self-reported health.
- The impact of being a female cancels out the positive associations observed for employment arrangements.
- Detrimental health factors on 50+ women have to be found principally outside of employment settings
